# Effects of three strength training methods on lower extremity strength, jump and sprint performance: a network meta-analysis

**DOI:** 10.3389/fphys.2025.1637520

**Published:** 2025-12-02

**Authors:** Shaohui Wang, Jie Zhen, Tao Xiao

**Affiliations:** School of Physical Education (Main Campus), Zhengzhou University, Zhengzhou, China

**Keywords:** olympic weightlifting, plyometric training, stretch-shortening cycle, strength, jump, sprint

## Abstract

**Background:**

Weightlifting (WT) and Plyometric training (PT) may lead to comparable enhancements in strength, jump, and sprint performance. However, these two training modalities appear to differ significantly in their primary focus and underlying mechanisms.

**Objective:**

Examining the differences between WT and PT in improving lower extremity sports performance.

**Methods:**

A systematic search was conducted from five databases, including Web of Science, PubMed, Scopus, Elsevier, and Springer. Two authors developed specific inclusion and exclusion criteria to screen relevant literature based on the study objectives. The quality of the included studies was assessed using the Physiotherapy Evidence Database (PEDro) scale. We conducted both direct comparisons and network meta-analysis on the eligible studies. The assumptions of similarity, homogeneity, and consistency within the Bayesian network were also confirmed.

**Results:**

A total of 17 studies met the inclusion criteria, involving 394 participants. All studies were found to have a low or moderate risk of bias, with average score of 4.29. The Bayesian network meta-analysis showed no significant differences. According to the SUCRA rankings, TT was most likely to excel in squat jumps (SJ) (SUCRA = 0.76) and maximum strength (SUCRA = 0.95), WT for sprint (SUCRA = 0.77), and PT for countermovement jumps (SUCRA = 0.76). The tests of similarity, homogeneity and consistency of the network meta-analysis were also generally valid. The funnel plot and Egger regression tests indicated no publication bias.

**Conclusion:**

In summary, the WT programs are more effective at improving sprint performance by increasing power, while the PT programs improve jumping performance by improving the stretch-shortening cycle.

**Systematic Review Registration:**

identifier CRD 420250540130.

## 1 Introduction

Lower extremity strength and power are foundational to athletic success across diverse sports, from explosive track and field events to dynamic team sports like basketball and soccer (Muehlbauer et al., 2015; Suchomel et al., 2016). These physical traits directly determine an athlete’s capacity to sprint, jump, and change direction rapidly—actions that often differentiate elite competitors (Young et al., 1995; Burnie et al., 2018; Young, 2006; Vosburg et al., 2022; Negra et al., 2020). Yet, despite widespread recognition of its importance, a critical question persists: What is the most effective training methodology to optimize lower-body power and translate it into measurable athletic outcomes?

Two dominant paradigms have emerged in strength and power training: weightlifting training (WT) and plyometric training (PT). Weightlifting exercises and their derivatives primarily emphasize the development of maximal strength and power through movements such as the snatch and clean and jerk, which involve lifting heavy loads with explosive actions (Morris et al., 2022; Young, 2006). This type of training enhances neuromuscular coordination, muscle hypertrophy, and the ability to generate force rapidly, making it particularly effective for athletes who require high levels of absolute strength and power (Simenz et al., 2005; Ebben, 2009). In contrast, PT focuses on improving the stretch-shortening cycle (SSC) efficiency, which is crucial for activities that involve rapid transitions from eccentric to concentric muscle actions, such as jumping and sprinting (Markovic et al., 2007; Watkins, 2009; Booth and Orr, 2016; Komi, 2000). Plyometric exercises, like depth jumps and bounding, aim to increase muscle-tendon stiffness and enhance the storage and release of elastic energy, thereby improving reactive strength and explosive performance (Markovic and Mikulic, 2010). While both modalities enhance power-related metrics, their mechanistic divergence—WT’s emphasis on absolute force versus PT’s SSC efficiency—suggests context-dependent efficacy (Morris et al., 2022; Berton et al., 2018). Determining the performance objectives under which WT or PT should be prioritized, and whether their benefits extend uniformly across key indicators such as vertical jump, sprint speed, and injury resilience, is essential for evidence-based training prescription.

Existing comparative analyses on the efficacy of different training modalities are largely inconclusive, hindering clear guidance for practitioners. For instance, earlier reviews such as those by (Arabatzi et al., 2010; Hackett et al., 2016) concluded that WT and PT did not demonstrate significant differences in improving jumping performance. Early meta-analyses, such as that by (Berton et al., 2018), while reporting advantages of WT over traditional resistance training (TT) for countermovement jump (CMJ) height, also found no significant differences between WT and PT. However, these conclusions were constrained by narrow outcome measures (e.g., CMJ alone) and limited sample sizes (*n* = 7 studies), failing to account for sport-specific demands or multi-dimensional performance metrics. Subsequent work by (Morris et al., 2022) expanded the evidence base, corroborating WT’s advantages over TT while reaffirming WT-PT equivalence—yet critical gaps persist. The robustness of these findings requires rigorous evaluation against a broader spectrum of athletic tasks, and methodological limitations in prior syntheses may obscure nuanced differences between WT and PT. Notably, conventional pairwise meta-analyses struggle to resolve these questions due to fragmented direct comparisons and heterogeneous study designs.

This uncertainty underscores the need for an analytical framework capable of synthesizing direct and indirect evidence across diverse interventions. Network meta - analysis (NMA) presents a powerful solution to overcome the limitations of traditional pairwise comparisons. NMA combines both direct and indirect evidence from multiple interventions within a unified analytical framework, enabling a more comprehensive comparison of different training methods. This approach allows for a more accurate estimation of the relative effectiveness of WT and PT, even when direct head - to - head comparisons are scarce. By synthesizing data from a broader range of studies, NMA can offer valuable insights into the comparative advantages of these training modalities for enhancing lower - body power and athletic performance. The primary aim of this study is to use network meta - analysis to systematically compare the effectiveness of weightlifting training and plyometric training in terms of lower - body power and athletic performance. Specifically, this study intends to: (1) Assess the relative impact of WT and PT on key measures of lower extremity power, such as vertical jump height, and sprint performance. (2) Investigate the differences in the emphases of three training modalities in enhancing sports performance. (3) Provide evidence-based recommendations for integrating WT and PT into sports training programs. By resolving longstanding controversies in strength programming, this analysis seeks to empower coaches, athletes, and sports scientists with data-driven insights for optimizing lower-body power development.

## 2 Methods

This systematic review and network meta-analysis has been designed and reported in accordance with the Preferred Reporting Items for Systematic Reviews and Meta-Analyses (PRISMA 2020 statement), the extended PRISMA statement for Network Meta-Analyses, and the Cochrane Handbook for Systematic Reviews of Interventions (Page et al., 2021; Shuster, 2011). The completed PRISMA 2020 checklist is available in Supplementary Material S1 *.* The protocol was previously registered in the international database of systematic reviews PROSPERO (ID: CRD420250540130, in April 2024).

### 2.1 Search strategy

A thorough literature search was carried out across multiple databases, namely Web of Science, PubMed (Pubmed Central), Scopus, Elsevier, and Springer. The search imposed no constraint on the publication date; however, it was confined to articles published in English. To pinpoint relevant studies, Boolean search logic was utilized, with specific search terms crafted around weightlifting training, plyometric training, traditional resistance training, and performance outcomes. The supplementary material holds a detailed account of the search strategy. Subsequently, two researchers independently went through the titles and abstracts of the identified studies to assess their relevance. Full - text versions of studies that appeared eligible were obtained and evaluated against the pre - set inclusion criteria. In cases where the two reviewers disagreed, discussions were held to iron out the differences.

### 2.2 Eligibility criteria

Inclusion criteria for the literature were established based on the PICOS (Participants, Interventions, Comparators, Outcomes, and Study Design) guidelines. Studies involving healthy male or female participants were selected without restrictions on publication dates.

The participants included in this systematic review were individuals who maintained good health throughout the experimental process, with no restrictions on ethnicity, nationality, or sex. Age restrictions are limited to early adulthood (18–30 years old). Studies were excluded if they involved participants with chronic diseases or if they included animal subjects, which were identified and removed during the title and abstract screening phase.

Studies must have compared at least two of the following training modalities: weightlifting training (WT), traditional resistance training (TT), or plyometric training (PT). The intervention period was required to be a minimum of 6 weeks, with at least two training session per week, totaling at least twelve sessions.

The control group performed resistance training or the same regular interventions as the intervention group. For the network meta-analysis, we defined comparison groups based on the interventions studied. In this network, TT was designated as the reference treatment to allow for the quantification of relative treatment effects between PT, WT, and TT. Studies included in the network either compared one of these interventions against TT, or compared PT directly against WT.

Studies were required to report on at least one performance measure, including lower-limb maximum strength (1RM squat), jumping performance (squat jump, countermovement jump), or sprint performance (short-distance sprint).

Only randomized controlled trials (RCTs) published in English were included. For studies based on the same sample, only one article was included to avoid duplication.

Exclusion criteria included duplicate publications, insufficient data availability despite attempts to contact authors, and studies with significant methodological flaws or poor testing protocols. Studies failing to meet the minimum quality standard of 3 points on the PEDro scale.

### 2.3 Data extraction

Adhering to the PRISMA guidelines for literature selection and data extraction, the data collection form is based on the extraction form modified from the “Cochrane Handbook for Systematic Reviews of Interventions” (Higgins et al., 2019). Extracted data included: (1) Basic study information (first author, year of publication). (2) Participant demographics (gender, age, sample size, subject identity). (3) Intervention details (training methods, frequency, load, duration, number of sessions). (4) Outcome measures (1RM squat, squat jump, countermovement jump, sprint). During the process of data extraction, data from all intervention groups is obtained. In instances where direct acquisition of data is not feasible, methodologies such as GetData Graph Digitizer and Cochrane data conversion tools are employed to ensure uniform integration of the data. To maintain rigor, literature lacking crucial data that cannot be converted or that does not meet the established criteria is excluded directly. For the same intervention measures within the same literature, a combined treatment is applied.

### 2.4 Methodological quality and publication bias

The methodological quality of the included studies was assessed using the Physiotherapy Evidence Database (PEDro) scale, which evaluates external validity (Item 1) and internal validity (Items 2–9), as well as the adequacy of statistical information (Items 10–11). Each criterion met scores 1 point, with a maximum possible score of 10 (excluding Item 1). Studies scoring 6–10 were considered high quality, 4-5 moderate quality, and ≤3 low quality. Two authors independently evaluated the methodological quality of each study, with discrepancies resolved through discussion and consultation.

Publication bias was assessed using Egger’s regression test and funnel plot asymmetry adjustments (Egger et al., 1997).

### 2.5 Statistical analysis

The Bayesian network meta-analysis model was chosen for its ability to rank interventions based on posterior probabilities. This approach overcomes the limitations of frequentist methods, which estimate parameters by iteratively maximizing the likelihood function. Such methods can lead to instability and biased results. In contrast, the Bayesian model is more flexible and is currently the preferred method in evidence-based research.

The analysis was conducted using R (version 4.3.3 with the gemtc package) and Stata (MP 18). Given the different measurement methods for the outcome indicators, a random effects model was chosen to minimize heterogeneity and determine the overall effect of the training interventions on the outcomes, including 1RM, SJ, CMJ, and SP.

To objectively and accurately reflect the relative effects of the three intervention methods and to exclude the influence of the conventional training control group on effect sizes, direct comparisons were made between the three interventions. The pre- and post-training changes in the experimental and control groups were used to calculate the standardized mean difference (SMD) and its 95% confidence interval (CI) for pooling effect sizes. Heterogeneity was assessed I^2^ statistic (low <25%, moderate 25%–50%, high >50%). Statistical significance was set at p < 0.05.

## 3 Results

**FIGURE 1:**
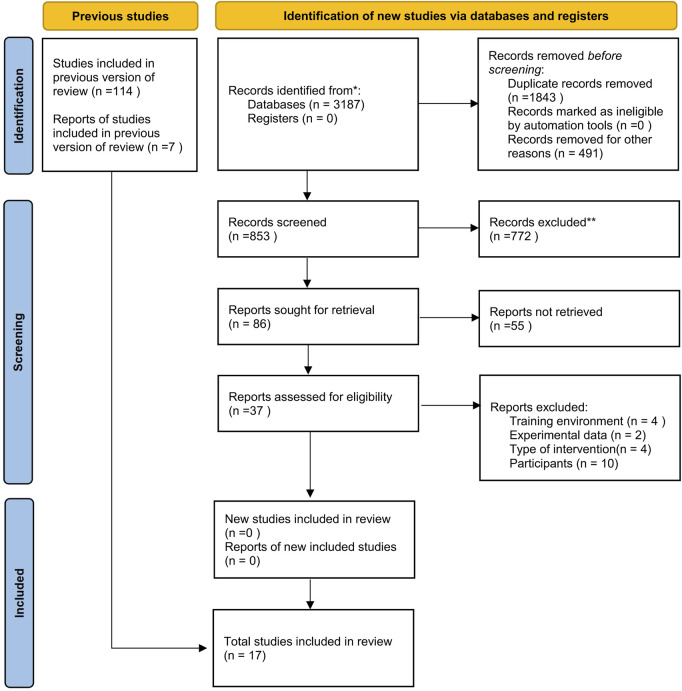
Flow diagram of literature screening and selection for the network meta-analysis.

A total of 3,187 references were initially searched from five databases, and an additional eight articles were obtained from reference sources. Duplicates were removed using the “Find Duplicate References” feature in EndNote, resulting in 853 unique papers. After screening titles, abstracts, and full texts, 86 articles were selected for further review. In the end, 17 pieces of literature were selected after a thorough full-text review (Arabatzi et al., 2010; Arabatzi and Kellis, 2012; Berton et al., 2022; Boraczyński et al., 2023; Ciacci and Bartolomei, 2018; Hawkins et al., 2009; Helland et al., 2017; Hoffman et al., 2004; Ita and Guntoro, 2018; MacDonald et al., 2012; Oranchuk et al., 2019; Ribeiro et al., 2020; Teo et al., 2016; Tricoli et al., 2005; Vissing et al., 2008; Whitehead et al., 2018; Wilson et al., 1993). The flow diagram of the literature is presented in Figure 1.

### 3.1 Characteristics of study

Ultimately, 17 studies were included in the analysis, comprising 15 two-arm trials and 2 three-arm trials. Among the 17 studies, there were a total of 394 participants distributed across 12 plyometric training groups (*n* = 134), 13 traditional resistance training groups (*n* = 142), and 11 Olympic weightlifting training groups (*n* = 118). The age range of participants was 18–30 years. The fundamental characteristics of the literature are presented in Table 1.

**TABLE 1:** Basic Characteristics of Subjects and Training Interventions.

Study(first author/years)	Intervention	Age	n	Sex	Exercise	Frequency (times/weeks)	Sessions	Volume (sets × reps)	Outcome	Scores (PEDro)
Arabatzi et al., 2010	PT	20.3	9	male	double-leg hurdle hops, alternated single-leg hurdle hops, double-leg hops, half-squat	3/8	24	1-2w: 4 × 6 68cm 3.15m 3-4w: 6 × 6 74cm 3.15m 5-8w: 6 × 6 74cm 2.7m	SJ, CMJ	4
	WT	20.3	9	male	power clean, snatch, clean and jerk, high pull			1-2w: 4 × 4 75%1RM 3-4w: 4 × 6 80%1RM 5-8w: 4 × 4 80%-90%1RM		
Arabatzi and Kellis, 2012	WT	20.3	9	male	power clean, snatch, clean and jerk, high pull	3/8	24	1-2w: 4 × 4 75%1RM 3-4w: 4 × 6 80%1RM 5-8w: 4 × 4 80%-90%1RM	SJ, CMJ	4
	TT	20.3	9	male	leg press, leg curl, leg extension, half-squat			1-2w: 4 × 4 75%1RM 3-4w: 4 × 6 80%1RM 5-8w: 4 × 4 80%-90%1RM		
Berton et al., 2022	PT	23.9	15	male	bounce drop, jump double-leg, hurdle hops, horizontal jumps	2/8	16	1-4w: 4 × 6/4 × 4/3 × 3 5-8w: 5 × 6/5 × 4/3 × 3+1 × 2	SJ, CMJ	5
	WT	24.1	15	male	high pull from the knee, power clean from the knee, mid-thigh clean pull			1-4w: 5 × 5/5 × 5/4 × 5 5-8w: 6 × 5/6 × 5/5 × 5		
Boraczyński et al., 2023	PT	22.5	15	male	leg lifts, plank kicks, leg flexes, leg kicks	2/8	16	1w: 2 × 8/2 × 8/2 × 15/2 × 15 2-7w: 3-4 × 6-12 8w: 6 × 7	SJ, CMJ, 1RMsquat, T10,30	4
	TT	22.1	15	male	half-squat, leg press, leg curl, leg extension lunges calf raises			1-3w: 3 × 10 80%1RM 4-6w: 3 × 8 85%1RM 7-8w: 3 × 6 90%1RM		
Ciacci and Bartolomei, 2018	WT	24.3	10	male	power clean	2/16	32	4 × 8	SJ, CMJ,	4
	TT	20.1	12	male	half-squat			4 × 8		
Hawkins et al., 2009	PT	21.5	10	male	jumps in place, squat jumps, a-skips, single-leg hops, double-leg hops	3/8	24	1-4w: 3 × 3-10 5-8w: 3 × 5-15	SJ, CMJ, 1RMsquat	5
	WT		10	male	hang clean, high pull, power push(front), snatch balance, front squats			1-4w: 3 × 5-8 5-8w: 3 × 2-6		
	TT		9	male	squat, lunges			1-4w: 3 × 6-10 5-8w: 3 × 4-8		
Helland et al., 2017	WT	20	13	male/ female	power clean, snatch, front squats, clean and jerk	2-3/8	21	2-5 × 5RM	SJ, CMJ, 1RMsquat, T30	5
	TT	20	13	male/ female	squat, lunges			2-3 × 5RM		
Hoffman et al., 2004	WT	19.3	10	male	clean (floor), clean pulls (above knee), push jerks, snatch pulls (floor), snatch pulls (above knee)	4 × 15	60	1-5w: 2-4 × 8 10RM 6-10w: 4-5 × 5-8RM 11-15w: 3-5 × 3-6RM	1RMsquat	5
	TT	18.9	10	male	squat, dead lift, leg extensions, leg curls, standing calf raises			1-5w: 3-4 × 8-10RM 6-10w: 3-4 × 6-8RM 11-15w: 3-5 × 4-6RM		
Ita and Guntoro, 2018	PT	21.3	15	male	skipping, rope jump, low jump and short stride, hops and jumping, jumping in top bench or rope	6 × 6	36		T30	4
	TT	21.3	15	male				2-5 × 2-5 75%-90%1RM		
MacDonald et al., 2012	PT	20.6	11	male	drop jump, box jump, lateral jump	2 × 6	12		1RMsquat	3
	TT	22	9	male	squat, dead lift, standing calf raise			3 × 3-6 75%-90%1RM		
Oranchuk et al., 2019	WT	19.6	9	male/ female	high pull, squat, clean and jerk	2/10	20	4 × 5-12 60%-75%1RM	SJ, CMJ	4
	TT		9	male/ female	squat, glute ham raise, trap-bar jump squat			4 × 5-12 60%-75%1RM		
Ribeiro et al., 2020	PT	18.6	8	male	squats, lunges and submaximal jump actions.	2/7	12	1-2w: 1-3 × 8 3-6w: 2-3 × 8-12 7-8w: 2-4 × 6-12 9-12w: 2-4 × 8-12	SJ, CMJ	4
	TT	18.4	8	male	half-squat, hip-thrust			1-4w: 4 × 8		
Teo et al., 2016	PT	24.2	13		double leg jumps, depth jumps, half-squat			1-3w: 4-8 × 4-10 4-6w: 6-8 × 4-12	T5, 20	5
	WT		13		hang power clean, power snatch, half-squat			1-3w: 4 × 4-6 70%1RM 4-6w: 6 × 4-6 70%1RM		
Tricoli et al., 2005	PT	22	8	male	double-leg hurdle hops, alternated single-leg hurdle hops, single-leg hurdle hops, 40-cm drop jump, half-squat	3/8	24	1-4w: 4-6 × 4 5-8w: 6-10 × 4	SJ, CMJ, 1RMsquat	5
	WT	22	7	male	power clean, high pull, clean and jerk, half-squat			3-4 × 4-6RM 4-6 × 4-6RM		
Vissing et al., 2008	PT	25.1	7	male	countermovement jumps, hurdle jumps, drop jump	3/12	36	3-4 × 8-12	CMJ	4
	TT	25.1	8	male	hamstring curl, knee extension, incline leg press			3-5 × 4-10		
Whitehead et al., 2018	PT	21.3	10	male	leg lifts, leg kicks, skip hops, skips	2/8	16	1-4 × 4-20	1RMsquat,T10	4
	TT		10	male	squat, leg press, leg curl, lunges calf raises, extension			3 × 8-12		
Wilson et al., 1993	PT	22.1	13	male	depth jump	2/10	20	3-6 × 6-10	SJ, CMJ, T30	4
	WT	23.7	13	male	weighted jump squats					
	TT	21.9	15	male	squat					

PT: plyometric training; WT: weightlifting training; TT: traditional resistance training; reps: repetitions; SJ: squat jump; CMJ: countermovement jump; 1RM: 1 repetition maximum. T5, 10, 20, 30 : 5, 10, 20, 30 meters sprint test.

### 3.2 Methodological quality and risk of bias

The methodological quality of the included studies was evaluated using the PEDro scale for physical therapy. Overall, the average PEDro score of 4.29 indicated that the included studies were of moderate quality as shown in Table 1.

**FIGURE 2:**
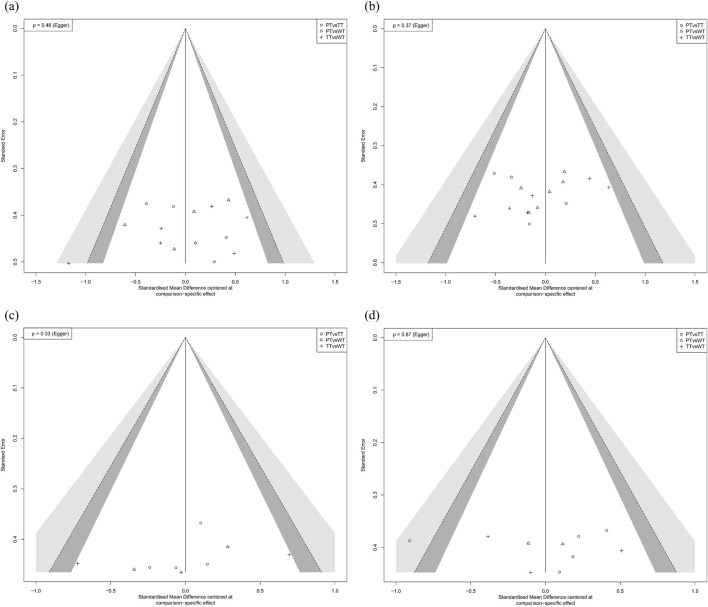
Funnel plot for various performance outcomes. Panels **(a−d)** present the risk of bias results for squat jump, countermovement jump, maximal strength, and sprint, respectively.

Of the various methods used to assess publication bias, funnel plots are most frequently employed due to their straightforward nature; however, analyzing publication bias with funnel plots necessitates subjective interpretation to detect bias and cannot objectively describe symmetry within the graphical representation. Egger’s tests and adjustments for asymmetry in funnel plots were utilized to assess publication bias. Funnel plot results suggested a generally symmetrical distribution, while Egger’s regression test results indicated no significant publication bias with p > 0.05, thus indicating the absence of publication bias. The findings from both the funnel plot and Egger’s regression tests confirmed that publication bias was largely absent from the studies (Egger et al., 1997). See Figure 2.

### 3.3 Meta-analysis

The outcome measures selected for the meta-analysis related to lower extremity strength performance were maximum strength, jumping ability, and sprinting ability. Lower limb maximum strength is a key indicator of athletes’ performance in both jumping and sprinting movements. The maximum strength measures included the highest weight achieved in one repetition of squat, half-squat, or leg press exercises. The short-distance sprints were defined by the time taken to complete sprints ranging from 5 to 30 m. Three types of standing vertical jump were assessed: squat jump, countermovement jump.

#### 3.3.1 Bayesian network meta-analysis

A network model was established for the network meta-analysis, which was then compiled using the Markov Chain Monte Carlo (MCMC) method. To ensure model convergence, we employed 20,000 pre-iterations and 50,000 actual iterations, a decision guided by established recommendations for MCMC analyses and preliminary checks demonstrating the stability of parameter estimates.

Model convergence was assessed using a combination of diagnostic tools. Trace plots and density plots (detailed in Supplementary Material S5) showed stable fluctuations with good overlap after 20,000 iterations, and the bandwidth in density plots approached and remained stable near zero, indicating effective convergence and saturation. Additionally, the potential scale reduction factor (PSRF), which measures the extent to which the posterior variance has shrunk relative to the prior variance, was used to further confirm convergence.

Model fit was assessed using the posterior mean residual (Dbar) and Deviance Information Criterion (DIC) to evaluate the model’s adequacy to the data.

#### 3.3.2 Testing for consistency

To examine the consistency assumption, a node-splitting method was employed to test one of the foundational assumptions of the network meta-analysis, producing outputs comparing effect sizes using only direct evidence, only indirect evidence, and all evidence combined. Discrepancies between direct and indirect evidence were assessed by examining the p-value column; a p-value of <0.05 for one or more comparisons indicates substantial inconsistencies within the network. A p-value of <0.05 for one or more comparisons indicates significant inconsistencies within the network. Analysis of the output results revealed that all tests for inconsistencies were not statistically significant, indicating a high level of consistency, thereby confirming the validity of the consistency tests for the network meta-analysis. For detailed results, see Supplementary Material S3 for the inconsistency analysis outcomes.

#### 3.3.3 Intervention comparisons

**FIGURE 3:**
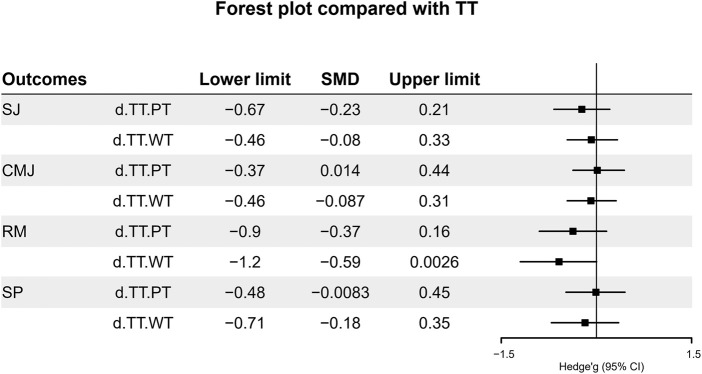
Forest plot compared with TT.

The Bayesian network model facilitated the merging of effect sizes from all studies, allowing for comparisons between PT and WT, using TT as the reference group. The results showed that among all outcome measures, only the maximum strength indicator displayed significance for traditional resistance training, with statistical relevance. Refer to Figure 3.

#### 3.3.4 SUCRA ranking

To identify the optimal intervention effects across different methodologies, SUCRA ranking probability analyses were conducted for each outcome measure, resulting in the creation of cumulative ranking charts. The outcomes of SJ, CMJ, and RM constituted positive effects, while the SP outcome was considered a negative effect, measured in seconds (s). The final outcomes revealed that TT yielded better improvements in squat jumps and maximum strength; PT showed more effectiveness for countermovement jump; and WT improves short-distance sprinting best. Specific rankings can be referenced in Table 2, which displays the SUCRA rankings.

**TABLE 2:** Probability of intervention ranking

Probability Outcome	PT	TT	WT	Ranking
SJ	0.1769	0.7602	0.5628	TT>WT>PT
CMJ	0.6148	0.5771	0.3081	PT>TT>WT
RM	0.4235	0.9488	0.1277	TT>PT>WT
SP	0.3718	0.3588	0.7694	WT>PT>TT

### 3.4 Heterogeneity analysis

The outcomes of the heterogeneity analysis indicated that the *I* ^*2*^ values for direct pairwise comparisons of the interventions across all outcome indicators were generally below 75%, thus reflecting low heterogeneity. Moreover, the network comparisons of the interventions also had an I^2^ value generally below 75% and was less than that of the direct comparisons. Please refer to Supplementary Material S4. for details on the heterogeneity analysis.

## 4 Discussion

This study aimed to investigate whether there are differences in the improvements of strength, jumping, and sprint between weightlifting training (WT) and plyometric training (PT). The network meta-analysis results indicated that WT has a greater advantage in improving sprint performance, traditional resistance training (TT) offers significant benefits in increasing maximal strength and squat jumps (SJ), and PT has a more pronounced effect on countermovement jump (CMJ) performance. However, these results were not statistically significant.

### 4.1 Plyometric training for lower extremity strength

PT has been widely recognized for its ability to enhance lower extremity strength, power, and sprint performance through its unique physiological mechanisms. The core principle of PT lies in the stretch-shortening cycle (SSC), which involves a rapid eccentric (lengthening) phase followed by an immediate concentric (shortening) contraction (Watkins, 2009; Markovic and Mikulic, 2010; Turner and Jeffreys, 2010; Tomalka et al., 2020). This mechanism allows muscles and tendons to store elastic energy during the eccentric phase, which is then released during the concentric phase, resulting in a more powerful and efficient movement. The SSC is particularly effective in improving sprint performance due to its ability to enhance the rate of force development (RFD) and reduce ground contact time, both of which are critical for explosive movements such as sprinting and jumping.

Physiological adaptations induced by PT include increased tendon stiffness and muscle fiber hypertrophy, particularly in type IIa fibers, which are responsible for fast-twitch muscle contractions (Komi, 2000; Mero and Komi, 1986). Studies have shown that PT can lead to a 24.1% increase in tendon stiffness, which enhances the efficiency of force transmission from muscles to bones, thereby improving power output during explosive movements (Fouré et al., 2010). Additionally, PT has been shown to increase the cross-sectional area (CSA) of key lower extremity muscles, such as the quadriceps and hamstrings, by up to 10%, further contributing to improved strength and power (Vissing et al., 2008). These adaptations are particularly beneficial for sprint performance, as they allow athletes to generate greater force in a shorter amount of time, which is essential for rapid acceleration and high-speed running.

The short ground contact time (<250 ms) characteristic of PT exercises is another key factor that makes it particularly effective for improving sprint performance (Malisoux et al., 2006; Watkins, 2009; Flanagan and Comyns, 2008). During sprinting, the ability to quickly transition from the eccentric to the concentric phase of muscle contraction is crucial for maintaining high running speeds. PT training enhances this ability by improving neuromuscular coordination and increasing the stiffness of the lower extremity muscles and tendons, which reduces the time required to generate force during each stride (Bouguezzi et al., 2020). This is why PT is often more effective than other training modalities for improving sprint-related performance.

In terms of practical applications, PT should be tailored to the athlete’s experience level and specific performance goals. For beginners, it is recommended to start with low-intensity exercises involving 40–80 ground contacts per session, gradually progressing to higher intensities as the athlete becomes more experienced (Jensen and Ebben, 2005). Elite athletes, on the other hand, may benefit from high-intensity PT sessions involving 200–400 ground contacts, as these can further enhance power output and sprint performance. Controversially, many studies have found that high-intensity plyometric training is highly susceptible to injury and fatigue (Tofas et al., 2008; Drinkwater et al., 2009; Chatzinikolaou et al., 2010). For instance, one study found that high-volume plyometric training (212 ground contacts) already severely impairs a muscle’s ability to generate force and slow evoked contraction velocity, even if the athlete doesn’t feel fatigued (Drinkwater et al., 2009). It is crucial to balance training intensity with adequate recovery, as excessive PT can lead to neuromuscular fatigue and muscle damage, particularly in type II fibers, which are highly susceptible to overtraining (Macaluso et al., 2012). In addition, an anonymous survey study showed that many practitioners routinely use significantly lower amounts of sessions than previously recommended in the literature (p < 0.05) (Watkins et al., 2021). Thus, to align with the practical need for safe, effective PT programming (and address the nuance of volume prescription across athlete populations), training design must prioritize individualized volume adjustment (rather than a one-size-fits-all range) and integrated recovery strategies.

In summary, the physiological mechanisms underlying PT, particularly the SSC, make it highly effective for improving sprint performance by enhancing tendon stiffness, muscle fiber hypertrophy, and neuromuscular coordination. To optimize its benefits, PT should be implemented with careful consideration of the athlete’s experience level and recovery needs, ensuring that training intensity is appropriately matched to the desired performance outcomes.

### 4.2 Weightlifting training for lower extremity strength

The weightlifting training methods, including the clean and jerk, snatch, and deadlift, facilitate the achievement of coordinated triple extension in the primary lower extremity joints (hip, knee, and ankle) during exercise. Weightlifting dates back to ancient China, Egypt, and Greece, and it wasn’t until the 1950s that it found its application in foundational training for non-weightlifting athletes involved in team sports and athletics (Comfort et al., 2023; Todd, 2008; Shurley and Todd, 2012). Since that time, weightlifting-style training methods have been extensively applied in strength training. Fry’s study revealed that weightlifters exhibit a greater proportion of type IIA fibers and a comparatively higher relative content of myosin heavy chain IIA sub than normal adults. Furthermore, weightlifting performance shows strong correlation with the percentage of type IIA fibers (r = 0.94) and the area percentage of type IIA fibers (r = 0.83) (Fry et al., 2003). Numerous cross-sectional studies have provided evidence that high-intensity training in weightlifters results in hypertrophy of type IIA fibers (Fry et al., 2003; Tesch et al., 1984). Longitudinal HIRE studies on non-weightlifters also indicate the potential conversion from IIX to IIA fiber types (Fry, 2004). Olympic weightlifting essentially relies on movements like the clean, jerk, snatch, and deadlift, stressing the quick movement and release of the, thus representing a dynamic and rapid form of training that allows athletes to generate substantial force and power in a brief timeframe; this dynamic and rapid training method effectively improves an athlete’s explosive strength. Similarly, jumping involves quickly moving the body vertically and ‘releasing’ it into the air. This attribute of weightlifting training overlaps with the biomechanics of sprinting and jumping. Extensive longitudinal studies and systematic evaluations have reported the efficacy of weightlifting training in enhancing athletic strength performance. In weightlifting training, the muscle rate of force development and power output are maximized, with numerous researchers discovering that weightlifting significantly enhances power during jumps (Arabatzi and Kellis, 2012; Hawkins et al., 2009; Ostrowski et al., 1997). Garhammer’s study additionally identified similarities between the force-velocity loading profiles of weightlifting training and the movement patterns involved in jumping (Garhammer, 1993). Collectively, these findings suggest that weightlifting training can more effectively improve rapid muscle strength and power output to conventional training methods. Indeed, while weight training greatly enhances lower extremity performance, it also places significant demands on the flexibility of the hip, ankle, and upper limb joints due to its specific movement patterns. People with inadequate joint flexibility and insufficient technical proficiency may fail to experience training benefits, thereby raising their injury risk during exercise. Elite level weightlifters engage in high-intensity resistance exercises (HIRE, ≥80% of 1RM) twice or more each day, accumulating 6-7 sessions a week (Garhammer, 1993; Thrush, 1995). In elite weightlifters, partitioning the predefined training volume into two sessions on the same day can markedly enhance muscle strength, hypertrophy, and maximal neural activation of the trained muscle groups (Häkkinen and Kallinen, 1994; Hartman et al., 2007). Nevertheless, as most exercises executed by weightlifters revolve around competitive lifting and similar multi-joint activities, the same primary muscle groups experience extensive contractions during each training session. Existing evidence further suggests that repeated HIRE targeting the same muscle groups can result in sustained suppression of critical synthetic metabolic mediators, prolonged inflammatory response, and decreased muscle performance (Fry et al., 1994b; Fry et al., 1994a; Fry et al., 2006; Crewther and Christian, 2010; Crewther et al., 2011). Compared to conventional resistance training, weightlifting may yield lower strength development; nevertheless, it demonstrates higher outputs in speed-strength performances in young athletes (Channell and Barfield, 2008). Moreover, the rate of force development (RFD) during weightlifting is considerably greater than that observed during squats (Chimera et al., 2004; Gruber et al., 2007; Young and Bilby, 1993). While there is no direct evidence suggesting adverse effects of weightlifting training on children or adolescents, there remains significant debate regarding its use among younger populations (Storey and Smith, 2012). Consequently, there is still a lack of well-defined physiological guidelines and age-appropriate weightlifting training recommendations.

### 4.3 Practical variations in lower extremity strength interventions among different training methods

Indeed, numerous studies suggest that traditional heavy resistance training primarily centers on maximizing muscle strength and muscle hypertrophy, which in turn enhances lower extremity performance, thus accounting for the substantial increases in maximal strength. On the other hand, weightlifting and plyometric training methods tend to reduce muscle hypertrophy due to their distinct movement patterns while focusing on enhancing lower extremity explosiveness (Cormie et al., 2011a; Cormie et al., 2011b; Folland and Williams, 2007; Komi, 1984). In exploring the impact of plyometric training, weightlifting training, and TT on performance improvement, the specificity of training methods must be addressed. Concerning training specificity, considerable evidence demonstrates that employing training methods analogous to the targeted movement patterns can result in greater transfer of training effects and subsequently yield better outcomes. Stone indicated that the SSC associated with plyometric training and the triple extension in weightlifting are evidently better aligned with the biomechanics of vertical jump performance (Stone et al., 2022). Additionally, many studies have confirmed the effectiveness of both plyometric training and weightlifting training in increasing power output (Cormie et al., 2011a; Cormie et al., 2011b; Suchomel et al., 2015). In addition, studies have revealed that weightlifting exercises and ultra-length exercises share similarities with the dynamics of jumping (including maximum strength and power, the timing to reach maximum strength and power, and relative strength and power) (Canavan et al., 1996; Haff et al., 2005; Haff et al., 2015). Although TT executes actions that resemble vertical jumps with similar hip, knee, and ankle extension movements, the application of higher loads (above 80% of 1RM) inevitably result in some loss of movement velocity (Cormie et al., 2007; Hedrick and Wada, 2008). Even under lower load conditions (45% 1RM), which are closer to practical movement speeds, TT displays a prominent deceleration phase. This deceleration phase is related to slower concentric velocities, diminished concentric forces, and decreased levels of muscle recruitment; these factors together result in a reduction in maximum power output. Another perspective argues that specificity in strength training is not a requirement; rather, an increase in muscle contractile strength will naturally enhance an athlete’s jumping capability. Supporters of this theory believe that the effectiveness of plyometric training and weightlifting in improving vertical jump performance is due to their ability to enhance muscle contractile capacity (Toumi et al., 2004; Adams et al., 1992). To summarize, while weightlifting and plyometric training may show somewhat less effectiveness in enhancing maximum muscle strength relative to TT, they appear to be more beneficial in improving quick strength. It is advised to integrate weightlifting methods and plyometric training with TT in training regimens.

## 5 Conclusion

This meta-analysis examined the differences in the effects of two similar training methods on improving lower extremity performance. Our findings affirm the superiority of resistance training in improving maximal strength. However, we observed that weightlifting and its derivatives were more effective in improving sprint performance, while plyometric training was more effective in enhancing countermovement jump. This is because the biggest feature of the WT approach is power enhancement, whereas PT focuses more on stretch-shortening cycle improvement. Therefore, this study suggests that although weightlifting training WT and PT share many similarities, they have different emphases.

Finally, practitioners should integrate these strength training modalities to mutually enhance the training effect, e.g., PT training after resistance training or PT exercises before WT to enhance neuromuscular performance, and these combinations are already widely used.
